# Prevalence and correlates of common mental disorder among HIV patients attending antiretroviral therapy clinics in Hawassa City, Ethiopia

**DOI:** 10.1186/s12991-019-0241-7

**Published:** 2019-09-03

**Authors:** Bereket Duko, Alemayehu Toma, Yacob Abraham

**Affiliations:** 1 Faculty of Health Sciences, College of Medicine and Health Sciences, Hawassa University, P. O. Box 1560, Hawassa, Ethiopia; 2 Faculty of Medical Sciences, College of Medicine and Health Sciences, Hawassa University, P. O. Box 1560, Hawassa, Ethiopia

**Keywords:** Common mental disorder, HIV, AIDS, South Ethiopia, Cross-sectional study

## Abstract

**Background:**

Common mental disorder (CMD) is a group of disorders which include depression, anxiety and somatoform disorders with significant contributions to the burden of disease. It can lead to high social, economic and individual costs because it accounts for one-third of the days missed at work and a fifth of all primary health-care appointment. This study was aimed to assess the prevalence and factors associated with common mental disorders among HIV patients in Hawassa City, Ethiopia, 2018.

**Methods:**

The cross-sectional study was conducted at Hawassa University Comprehensive Specialized Hospital, Ethiopia, among 294 HIV patients who were recruited through systematic sampling techniques. Common mental disorder was assessed through face to face interviews by trained professional psychiatry nurses using a WHO-validated 20-item version of the Self-Reporting Questionnaire (SRQ-20). Other possible risk factors of CMD were assessed using a structured questionnaire, perceived HIV stigma scale and Oslo Item 3 Social Support Scale.

**Results:**

A total of 294 HIV patients participated in the study giving a response rate of 98.7%. The mean (± SD) age of the respondents was 35.86 years (± 9.23). Among the study participants, being female [AOR = 1.25, (95% CI 1.01, 2.43)], being widowed [AOR = 1.99, (95% CI 1.51, 5.28)], having poor social support [AOR = 2.44, (95% CI 1.33, 4.51)], having previous history of psychiatric illness [AOR = 3.83, (95% CI 1.89, 9.33)] and HIV-related perceived stigma [AOR = 1.97, (95% CI 1.63, 2.89)] were more likely to have common mental disorder when compared to their counterparts.

**Conclusion:**

The prevalence of common mental disorder was high. The Ministry of Health should develop a guideline which helps to screen and treat common mental disorders at ART clinics. Further interventional research on risk factors of common mental disorder should be conducted to strengthen and broaden the current findings.

## Background

Common mental disorder (CMD) is a group of disorders which includes depression, anxiety and somatoform disorders with significant contributions to the burden of disease and disability in low- and middle-income countries [1]. It is believed that common mental disorder can lead to high social, economic and individual costs because they account for one-third of the days missed at work and a fifth of all primary health-care appointments [2]. The WHO in 2017 estimated that 4.4% and 3.6% of the global population suffered from depression and anxiety, respectively, while depression alone accounts for 5.4% in the African region [3].

CMD is highly prevalent in people living with HIV/AIDS (PLWHA) [4]. There is an interdependence and vicious circularity between mental health and HIV/AIDS. Study findings from the developed countries show that just under half of all PLHWA have a diagnosable mental disorder and in some instances a threefold higher rate of mental disorder [5]. Where mental illness and HIV co-occur; there is increasing evidence that the progression of the virus is greater and there is poor adherence to medication [4, 5].

PLWHA have a higher prevalence of common mental disorders than non-HIV-infected individuals [6]. Common mental disorder is among the most prevalent conditions with a prevalence of over 30% among PLWHA reported across studies in some low- and middle-income countries (LMIC), particularly for depression [7]. It contributes significantly to poor HIV disease outcomes such as increased HIV treatment failure and increased risk of HIV acquisition especially in LMIC [8].

HIV/AIDS imposes a major psychological burden to the infected individuals. People with HIV often suffer from common mental disorder, as they adjust to the impact of the diagnosis of being infected and face the difficulties of living with a chronic life-threatening illness, for example, shortened life expectancy, complicated therapeutic regimens, stigmatization and loss of social support. HIV infection can be associated with high risk of suicide [9].

Provoking factors for common mental disorder in PLWHA are related to stress, low social support, number of negative life events, not disclosing HIV status and CD4 cell count of < 500 cells/mm^3^ [10–14]. Despite the fact that developing countries carry more than 90% of the burden of HIV/AIDS, little information about the interaction between HIV/AIDS and mental health is available from low- and middle-income countries [4–8]. In low- and middle-income countries, where specialists for mental health care are scarce, less specialized providers can be used to effectively deliver evidence-based treatments for common mental disorder. Therefore, with the aim of filling the knowledge gap, we assessed the prevalence of common mental disorder and associated factors among HIV-infected patients receiving antiretroviral treatment in Ethiopia.

## Methods

### Study setting and population

A hospital-based cross-sectional study design was employed at Hawassa University Comprehensive Specialized Hospital, Hawassa, Ethiopia. A total of 294 HIV patients who had regular follow-up at the antiretroviral therapy (ART) clinic were recruited for this study. From the study participants who had known psychiatric illness that hinders their capacity to participate in the study, those patients who were in the intensive care unit and those unable to communicate due to hearing problems were excluded from the study. Study participants were included using systematic sampling technique.

### Data collection

Professional psychiatry nurses who had taken all the necessary research training had collected the data using pretested interviewer-administered questionnaire. The data collection tool had socio-demographic characteristics, substance use-related questionnaire, clinical-related questionnaires, Oslo Social Support Scale, perceived HIV stigma scale and Self-Reporting Questionnaire 20 (SRQ 20). Social support was estimated using Oslo 3-Item Social Support Scale. This scale has the sum score scale ranging from 3 to 14 with three broad categories: “poor support” 3–8, “moderate support” 9–11 and “strong support” 12–14. This scale has not been validated to Ethiopian context; however, it was highly reliable in our pre-test with Cronbach’s *α* = 0.91 [15]. HIV-related perceived stigma was collected using an 11-item HIV stigma scale. This scale consisted of four-point Likert scale questions concerning perceived isolation, shame, guilt and disclosure of the HIV status. The item scores of the stigma questions were summed to construct a single stigma variable. Finally, the study participants were classified as having or not having perceived stigma using the mean of perceived HIV stigma (patients who scored greater than or equal to mean (≥ 19.21 or ≥ 5.97) [16, 17]. This instrument was adapted and translated to Amharic language and back to English. This scale also has not been validated to Ethiopian context; however, it was highly reliable in the study (Cronbach’s *α* = 0.95). The presence of common mental disorder was assessed using the 20-item version of the Self-Reporting Questionnaire (SRQ-20). It was developed by the World Health Organization (WHO) as a screening tool for common mental disorders [18]. The SRQ-20 has been tested in numerous settings and depending on the setting, community surveys or primary care, varied cutoff points have been used although a cutoff point of 7/8 is widely used. The patient’s psychiatric status has to be confirmed by a more extensive psychiatric interview. The questionnaire has already been translated into a variety of languages to allow it to be used among people of different cultures. Where SRQ-20 has been validated in other sub-Saharan countries, the optimal cutoff point for defining cases for CMD has also varied widely from ≥ 4 in Sudan to ≥ 10 in South Africa. Population surveys in Ethiopia have used various cutoff points to define cases of CMD, ≥ 6 in Addis Ababa and ≥ 11 in two rural settings, none of which were supported empirically. In the current study, CMD was measured using the locally validated Self-Reported Questionnaire (score of ≥ six indicating high levels of CMD). The SRQ-20 has previously been translated into Amharic and validated in Ethiopia, and it has been used for community surveys [19, 20].

### Data processing and analyses

We used EPI info version 7 for data entry and SPSS version 22 for data analysis. Multivariable logistic regression analysis was used to see the association of each independent variable with the variable of outcome and to identify potential confounders. A *p* value of less than 0.05 was considered statistically significant, and adjusted odds ratio with 95% CI were calculated to determine the association. Finally, data were presented by using numbers, frequencies, tables, OR and AOR.

## Results

### Socio-demographic characteristics of the study participants

A total of 294 HIV patients participated in the study, giving a response rate of 98.7%. The mean (± SD) age of the respondents was 35.86 years (± 9.23). All the study participants were Ethiopians. Regarding the religious view of the respondents, 126 (42.9%) were Orthodox religion followers, 104 (35.4%) were Protestant religion followers, 45 (15.3%) were Muslims and 19 (6.5) were Catholic religion followers (Table 1).

**Table 1:** Frequency distribution of socio-demographic and socioeconomic factors among HIV patients at Hawassa, Ethiopia, 2018 (*n* = 294)

Variable	Category	Frequency (*n*)	Percent (%)
Gender	Male	120	40.8
	Female	174	59.2
Age	18–29	54	18.4
	30–39	116	39.5
	40–49	83	28.2
	≥ 50	41	13.9
Marital status	Single	68	23.1
	Married	145	49.3
	Divorced	39	13.3
	Widowed	42	14.3
Religion	Muslim	45	15.3
	Orthodox	126	42.9
	Protestant	104	35.4
	Catholic	19	6.5
Education	No formal education	95	32.3
	Primary school	77	26.2
	High school	36	12.2
	College/university	42	29.3
Occupational status	Housewives	44	15.0
	Civil servant	76	25.9
	Non governmental	25	8.5
	Daily labor	22	7.5
	Merchant	103	35.0
	Unemployed	24	8.2
Living status	Family	188	63.9
	Alone	58	19.7
	With relatives	48	16.3
Monthly income	< 2500 ETB	79.2	79.6
	2500–5000 ETB	14.6	14.6
	> 5000 ETB	5.8	5.8

### Clinical and psychosocial characteristics of the study participants

Regarding the clinical and psychosocial factors, 180 (61.2%) of the study participants had good drug adherence, 37 (12.6%) had family history of mental illness, 165 (56.1%) had HIV-related perceived stigma, 17 (7.1%) had comorbid tuberculosis infection and 31 (10.5%) were currently using substances (alcohol and tobacco products) (Table 2).

**Table 2:** Clinical and social support factors among HIV patients at Hawassa, Ethiopia, 2018 (*n* = 294)

Clinical and social support characteristics	Frequency	Percent %
Drug adherence		
Good	180	61.2
Poor	114	38.8
HIV status of the partner		
Positive	165	56.1
Negative	50	17.0
Do not know	79	26.9
Social support		
Poor	87	29.6
Good	207	70.4
Duration of the illness (years)		
< 5	89	30.3
5–10	150	51.0
≥ 10	55	18.7
CD4 cell count of the participants		
200	27	9.2
200–1000	259	88.1
≥ 1000	8	2.7
Family history of mental illness		
Yes	37	12.6
No	257	87.4
Previous history of mental illness		
Yes	17	5.8
No	277	94.2
Perceived stigma		
Yes	165	56.1
No	129	43.9
Social support		
Poor	87	29.6
Good	207	70.4
Comorbid illness		
TB	21	7.1
Heart diseases	9	3.1
Liver diseases	8	2.7
Diabetes	12	4.2
No comorbidity	244	82.9
Substance use (alcohol and cigarette)		
Yes	31	10.5
No	263	89.5

### Prevalence of common mental disorders and associated factors

The prevalence of common mental disorder in the current study was 32.7%. Binary logistic regression analysis showed that being female, being widowed, having perceived HIV-related stigma, having previous history of mental illness and those who had poor social support were significantly associated with common mental disorder (Table 3).

**Table 3:** Factors associated with common mental disorders among HIV patients attending hospitals at Hawassa, Ethiopia (*n* = 294)

Characteristics	Common mental disorder		COR at 95% CI	AOR at 95% CI
	Yes	No		
Age in years				
18–29	15	39	1.59 (0.59, 4.21)	
30–39	50	66	3.13 (1.33, 7.35)	
40–49	23	60	1.58 (0.64, 3.93)	
≥ 50	8	33	1	
Gender				
Male	107	91	1	1
Female	67	29	1.96 (1.17, 3.29)	1.25 (1.01, 2.43)**
Marital status				
Single	27	41	1	1
Married	26	119	0.33 (0.17, 0.93)	0.28 (0.12, 1.03)
Divorced	17	22	1.17 (0.53, 2.61)	0.82 (0.37, 2.06)
Widowed	26	16	2.47 (1.12, 5.44)	1.99 (1.51, 5.28)*
Living status				
Family	49	139	1	
Alone	30	28	3.04 (1.65, 5.59)	
Relatives or others	17	31	1.55 (0.79, 3.06)	
HIV status of partner				
Positive	44	121	0.56 (0.32, 0.99)	
Negative	21	29	1.21 (0.54, 2.31)	
Do not have partner	31	48	1	
Social support				
Poor	45	42	3.28 (1.94, 5.55)	2.44 (1.33, 4.51)**
Good	51	156	1	1
Previous history of mental illness				
Yes	11	6	4.14 (1.48, 11.56)	3.83 (1.89, 9.33)*
No	85	192	1	1
Family history of mental illness				
Yes	8	29	0.53 (0.23, 1.21)	
No	88	169	1	
Perceived HIV stigma				
Yes	55	74	2.25 (2.13, 3.73)	1.97 (1.63, 2.89)*
No	41	124	1	1
CD4 cell count				
< 200	11	16	2.06 (0.35, 12.17)	
200–1000	83	176	1.42 (0.54, 7.16)	
≥ 1000	2	6	1	

**p < 0.01, *p < 0.05

## Discussion

This study was conducted to assess the prevalence and factors associated with common mental disorder in HIV-positive patients who were enrolled into the ART program in Ethiopia. The prevalence of common mental disorder was 32.7%, which was lower than that of other studies conducted in three hospitals in Ethiopia [21, 22], Nigeria [23] and Uganda [24]. On the other hand, the current finding was higher than study’s findings from Debremarcos, Ethiopia [25], Dilla, Ethiopia [10], and South Africa [19]. The variation in prevalence might be attributed to the difference in the following factors. The first variation is attributed to the difference in the data collection tools which were used to measure common mental disorder. Some studies used Kessler Psychological Distress Scale (K-6 and 10), General Health Questionnaire (GHQ-10) and Self-Reporting Questionnaire (SRQ-20) with lower or higher cutoff point. Secondly, the study population and sample size discrepancy might play a great role in the variation. For example, a study conducted in three hospitals in Ethiopia included TB/HIV co-infected patients which might overestimate the magnitude of CMD [21, 22], while other studies included a large sample size. The study setting and design (case–control vs. cross-sectional design) also contributed to the mentioned difference. The majority of the studies were conducted in the hospital setting, while others in the community setting.

Females were 1.25 times more likely to have common mental disorder when compared to males. Findings from different studies revealed that common mental disorders such as anxiety, depression and somatoform disorders are commonly seen in females, which might be attributable to the biological difference between both sexes [26].

Common mental disorder was significantly higher in divorced individuals. This is in line with other findings in Ethiopia [25] and South Africa [19]. This might be because the lack of emotional support from the partner might predispose them to this disorder. On the other hand, having mental illness could hinder the marital partner from handling the relationship and might lead them to divorce.

HIV-positive individuals who have reported HIV-related perceived stigma were 1.99 times more likely to have common mental disorder when compared to their counterparts. This is in agreement with other study findings [10, 19, 21, 25, 27, 28]. Individuals with perceived stigma might have poor self-image and be socially isolated from others and this in turn might predispose them to common mental disorders.

Patients having a previous history of psychiatric illness were more likely to have CMD. Although it was not clear whether the presence of HIV has an effect on the severity of previous psychiatric symptoms of patients, HIV patients with previous history of psychiatric disorders are prone to relapse. This might be because the chronicity of the disease may cause more severe symptoms and precipitate the relapse of previous mental illness [29, 30].

In this study, poor social support is an independent contributing factor for the development of common mental disorder. This might be because social isolation by HIV patients itself reduces social support that can result in a negative impact on their physical and mental well-being. This is also supported by the fact that these patients might prefer to avoid seeking help from others, and in addition social stigma towards them could increase their isolation and loneliness [29–31].

## Conclusion

The prevalence of common mental disorder was high. Being of female gender, being divorced, having perceived HIV-related stigma, having previous history of psychiatric illness and poor social support had significant association with common mental disorder. Health-care providers who work in HIV clinics should give more emphasis to female, divorced and patients with previous history of psychiatric illness. Delivering health education is also recommended for patients with HIV-related perceived stigma and poor social support. The Ministry of Health should develop a guideline which helps to screen and treat common mental disorder at ART clinics. Further interventional research on risk factors of common mental disorder should be conducted to strengthen and broaden the current findings.

## Limitation of the study

HIV-related perceived stigma scale and Oslo Social Support Scale have not been validated for the country of origin of the investigated sample. This may over- or underestimate the characteristics measured by these scales.

## Data Availability

All relevant data are in the paper. If further data are needed, kindly contact the principal author at berkole.dad@gmail.com.

## References

[1] Lopez DA, Mathers DC, Ezzati M, Jamison TD, Murray JLC (2006). Global burden of disease and risk factors.

[2] Fortes S, Villano LAB, Lopes CS (2008). Nosological profile and prevalence of common mental disorders of patients seen at the Family Health Program (FHP) units in Petrópolis, Rio de Janeiro. Rev Bras Psiquiatr..

[3] WHO. Depression and other common mental disorders global health estimates. 2017. https://apps.who.int/iris/bitstream/handle/10665/254610/WHO-MSD-MER-2017.2-eng.pdf. Accessed 23 Jan 2018.

[4] Chibanda D, Cowan F, Gibson L, Weiss HA, Lund C (2016). Prevalence and correlates of probable common mental disorders in a population with high prevalence of HIV in Zimbabwe. BMC Psychiatry..

[5] Ford N, Shubber Z, Meintjes G, Grinsztejn B, Eholie S, Mills EJ (2015). Causes of hospital admission among people living with HIV worldwide: a systematic review and meta-analysis. Lancet HIV..

[6] Brandt R (2009). The mental health of people living with HIV/AIDS in Africa: a systematic review. Afr J AIDS Res AJAR..

[7] Abas M, Broadhead JC, Mbape P, Khumalo-Sakatukwa G (1994). Defeating depression in the developing world: a Zimbabwean model. Br J Psychiatry.

[8] Mayston R, Kinyanda E, Chishinga N, Prince M, Patel V (2012). Mental disorder and the outcome of HIV/AIDS in low income and middle-income countries: a systematic review. AIDS.

[9] WHO. HIV/AIDS and Mental health report by secretariat EB124/6. https://apps.who.int/iris/handle/10665/2107. Accessed 27 Jan 2018.

[10] Tesfaye SH, Bune GT (2014). Generalized psychological distress among HIV-infected patients enrolled in antiretroviral treatment in Dilla University Hospital, Gedeo zone, Ethiopia. Glob Health Action..

[11] Narayan KM, Miotti PG, Anand NP, Kline LM, Harmston C, Gulakowski R (1999). HIV and noncommunicable disease comorbidities in the era of antiretroviral therapy: a vital agenda for research in low- and middle-income country settings. J Acquir Immune Defic Syndr.

[12] Leserman J, Jackson ED, Petitto JM, Golden RN, Silva SG, Perkins DO (1999). Progression to AIDS: the effects of stress, depressive symptoms, and social support. Psychosom Med.

[13] Ngum PA, Fon PN, Ngu RC, Verla VS, Luma HN (2017). Depression among HIV/AIDS patients on highly active antiretroviral therapy in the southwest regional hospitals of Cameroon: a cross-sectional study. Neurol Ther.

[14] Halman M, Carusone SC, Stranks S, Schaefer-McDaniel N, Stewart A (2014). Complex care needs of patients with late-stage HIV disease: a retrospective study. AIDS Care.

[15] Dalgard OS, Dowrick C, Lehtinen V, Vazquez-Barquero JL, Casey P, Wilkinson G (2006). Negative life events, social support and gender difference in depression. Soc Psychiatry Psychiatr Epidemiol.

[16] Bhate MS, Munjal S (2014). Prevalence of depression in people living with HIV/AIDS undergoing ART and factors associated with it. J Clin Diagn Res..

[17] Van Rie A, Sengupta S, Pungrassami P, Balthip Q, Choonuan S, Kasetjaroen Y (2008). Measuring stigma associated with tuberculosis and HIV/AIDS in southern Thailand: exploratory and confirmatory factor analyses of two new scales. Trop Med Int Health..

[18] Brian WP (2009). The impact of mental health and traumatic life experiences on antiretroviral treatment outcomes for people living with HIV/AIDS. J Med Antimicrob Chemother.

[19] Myer L, Smit J, Roux LL, Parker S, Stein DJ, Seedat S (2008). Common mental disorders among HIV-infected individuals in South Africa: prevalence, predictors, and validation of brief psychiatric rating scales. AIDS Patient Care STDS.

[20] Youngmann R, Zilber N, Workneh F, Giel R (2008). Adapting the SRQ for Ethiopian populations: a culturally-sensitive psychiatric screening instrument. Transcult Psychiatry.

[21] Deribew A, Tesfaye M, Hailmichael Y, Apers L, Abebe G, Duchateau L (2010). Common mental disorders in TB/HIV co-infected patients in Ethiopia. BMC Infect Dis.

[22] Deribew A, Deribe K, Reda AA, Tesfaye M, Hailmichael Y, Maja T (2013). Do common mental disorders decline over time in TB/HIV co-infected and HIV patients without TB who are on antiretroviral treatment?. BMC Psychiatry.

[23] Sulyman D, Abiodun OA, Yussuf AD (2013). Detection of psychiatric disorders by physicians attending to people living with HIV//AIDS in a Nigeria university teaching hospital. J Biol Agric Healthc.

[24] Harry P, Jed B, Emilio O (2005). Psychiatric disorders in HIV-positive individuals in urban Uganda. Psychiatr Bull.

[25] Zewdu S, Abebe N (2015). Common mental disorder among HIV infected individuals at comprehensive HIV care and treatment clinic of Debre Markos Referral Hospital, Ethiopia. J AIDS Clin Res.

[26] Bahrami F, Yousefi N (2011). Females are more anxious than males: a metacognitive perspective. Iran J Psychiatry Behav Sci.

[27] World Health Organization (2008). Mental health & HIV/AIDS therapy series.

[28] Perlick DA, Rosenheck RA, Clarkin JF, Sirey JA, Salahi J, Struening EL (2001). Stigma as a barrier to recovery: adverse effects of perceived stigma on social adaptation of persons diagnosed `with bipolar affective disorder. Psychiatr Serv.

[29] Nüesch R, Gayet-Ageron A, Chetchotisakd P, Prasithsirikul W, Kiertiburanakul S, Munsakul W (2009). The impact of combination antiretroviral therapy and its interruption on anxiety, stress, depression and quality of life in Thai patients. Open AIDS J..

[30] Berhe H, Bayray A (2013). Prevalence of depression and associated factors among people living with HIV/AIDSI in Tigray, Ethiopia. North Ethiopia: a cross sectional hospital based study. IJPSR..

[31] Prabha SC, Geetha D, Sanjeev R (2005). HIV & psychiatric disorders. Indian J Med Res.

